# Mapping and Validating a Point Neuron Model on Intel's Neuromorphic Hardware Loihi

**DOI:** 10.3389/fninf.2022.883360

**Published:** 2022-05-30

**Authors:** Srijanie Dey, Alexander Dimitrov

**Affiliations:** Department of Mathematics, Washington State University, Vancouver, WA, United States

**Keywords:** neuromorphic computing, LIF models, neural simulations, validation, performance analysis

## Abstract

Neuromorphic hardware is based on emulating the natural biological structure of the brain. Since its computational model is similar to standard neural models, it could serve as a computational accelerator for research projects in the field of neuroscience and artificial intelligence, including biomedical applications. However, in order to exploit this new generation of computer chips, we ought to perform rigorous simulation and consequent validation of neuromorphic models against their conventional implementations. In this work, we lay out the numeric groundwork to enable a comparison between neuromorphic and conventional platforms. “Loihi”—Intel's fifth generation neuromorphic chip, which is based on the idea of Spiking Neural Networks (SNNs) emulating the activity of neurons in the brain, serves as our neuromorphic platform. The work here focuses on Leaky Integrate and Fire (LIF) models based on neurons in the mouse primary visual cortex and matched to a rich data set of anatomical, physiological and behavioral constraints. Simulations on classical hardware serve as the validation platform for the neuromorphic implementation. We find that Loihi replicates classical simulations very efficiently with high precision. As a by-product, we also investigate Loihi's potential in terms of scalability and performance and find that it scales notably well in terms of run-time performance as the simulated networks become larger.

## 1. Introduction

The human brain is a rich complex organ made up of numerous neurons and synapses. Replicating the brain structure and functionality in classical hardware is an ongoing challenge given the complexity of the brain and limitations of hardware. The advent of supercomputers now allows for complex neural models, but at a huge cost of both software complexity and energy consumption.

A recent intense focus on brain studies, with the BRAIN initiative at the US (Insel et al., 2013), the Human Brain Project (HBP) in Europe (Markram et al., 2011), and philanthropic endeavors like Janelia Research Campus (Winnubst et al., 2019), and the Allen Institute for Brain Science (AIBS) (Lein et al., 2007), has produced a wealth of new data and knowledge, from records of neuronal and network dynamics, to fine-grained data on network micro- and nano-structure, bringing in the era of big neural data. At the same time, advances in electronics and the search for post-von Neumann computational paradigms has led to the creation of neuromorphic systems like Intel's Loihi (Davies et al., 2018), IBM's TrueNorth (Akopyan et al., 2015; DeBole et al., 2019; Löhr et al., 2020) and HBP's SpiNNaker (Khan et al., 2008), and BrainScaleS (Grübl et al., 2020).

Neuromorphic chips, as the name suggests—“like the brain”—can mimic the brain's function in a truer sense as their design is analogous to the brain (Thakur et al., 2018; Roy et al., 2019). Inspired by its architecture, we work on developing a principled approach toward obtaining simulations of biologically relevant neural network models on a novel neuromorphic commercial hardware platform.

Computers today are limited in this respect because of the way they have been built historically and the way they process data leading toward more energy and resource consumption in order to maintain versatility (Nawrocki et al., 2016; Ou et al., 2020). Neuromorphic chips on the other hand claim to be faster and more efficient for a set of specialized tasks (Bhuiyan et al., 2010; Sharp and Furber, 2013). In this study, we lay out a numeric groundwork to validate this assertion based on neural models derived from the primary visual cortex (VISp) of the mouse brain, as seen in recent work done on SpiNNaker (Knight and Furber, 2016; Rhodes et al., 2019). Intel's neuromorphic chip “Loihi” serves as our neuromorphic platform. Results obtained in Loihi are validated against classical simulations (Rossant et al., 2010; Nandi et al., 2020; Wang et al., 2020) given by AIBS's software package the Brain Modeling Toolkit (BMTK) (Dai et al., 2020).

In this manuscript we focus on the Loihi architecture, as it is at present one of the most powerful platforms with specialized digital hardware and significant software support. While TrueNorth has a similar combination of hardware and programming support, its inter-neuron connectivity capability is relatively limited; Loihi approaches the human-scale connectivity density of interest to our research. SpiNNaker has similar capabilities, but is constructed of standard CPU hardware. Loihi's capabilities on the other hand, are built-in on a chip, thus forcing us to explore new programming paradigms. And recent and current state of the art hybrid analog-digital platforms, like Neurogrid (Benjamin et al., 2014), Braindrop (Neckar et al., 2019), DYNAP-SE2 (Moradi et al., 2017), and BrainScaleS(2) (Pehle et al., 2022) are beyond the scope of this manuscript. However, we believe that the simulation and programming paradigms developed on the Loihi platform can generalize to these analog platforms as well, and thus decrease the development time on these unfamiliar architectures.

**Figure 1:**
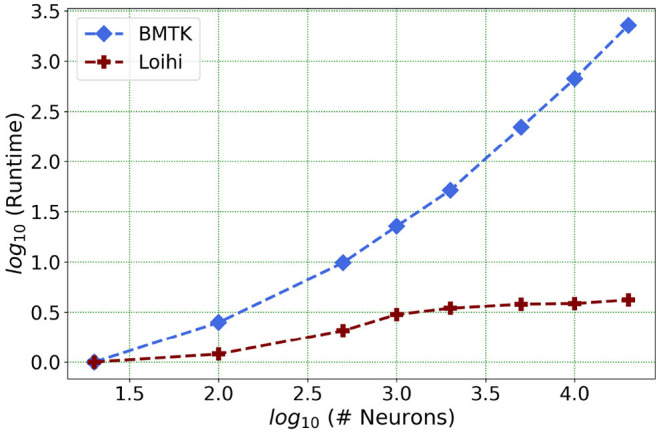
As the network size increases, Loihi outperforms consistently in terms of time. The figure shows runtime comparison of 500 *ms* of dynamics for up to 20,000 neurons for Loihi and BMTK, with the values scaled by the respective smallest runtime. Loihi has a maximum runtime of up to 12 ms, whereas BMTK runtime goes up to 273 s (See **Table 3** for the explicit runtime values and Section 4.4 for further details about the network.).

We present one of our main motivations for this project in Figure 1, which highlights Loihi's advantage in performance when compared to standard simulations. Overall, our initial implementation indicates that Loihi is quite efficient in terms of compute-time in context of large brain network simulations and thus shapes our central motivation for this work (see Figure 1 and **Table 3**). This manuscript mainly focuses on the trade-offs necessitated by these implementations, that is, how precise are the Loihi simulations when validated against BMTK simulations, given their very different hardware and programming architectures?

As a starting point, we focus on a class of neural network building blocks: point neuronal models as used in large AIBS simulations of biological neural networks. We do so because the Generalized Leaky Integrate and Fire Models (GLIFs, Teeter et al., 2018) have been found to be appropriate for reproducing cellular data under standardized physiological conditions. The data used for this study is made available by the AIBS (AIBS, 2020).

The paper is organized as follows. In Section 2, we describe in detail the features of Loihi and the differences between the neuromorphic and classical hardware that form the basis for this study. Section 3 explains the implementation of the continuous LIF equation on classical computational architecture using BMTK vs. the discrete Loihi setting. Also, we list the validation methods and the cost function that is used to draw comparisons between the implementations. In Section 4, we list out and explain the various results leading to a qualitative and quantitative assessment between the two platforms based in part on methods from Gutzen et al. (2018). Finally, Section 5 lays the ground for future work with expected improvements based on the second generation of the Loihi chip, Loihi 2 (Intel, 2022).

## 2. Comparison Between Classical and Neuromorphic Platforms

At present, various simulators are available for implementing spiking neural networks (Brette et al., 2008). In this section, we lay out the details of the mathematical model and the platforms we use for our work. For the classical simulation, we use the Brain Modeling Toolkit (BMTK) (Dai et al., 2020) developed by the AIBS. Being open source, these resources enable us to experiment with a varied range of data and thus support our extensive validation of neuronal models in Loihi. Intel's fifth-generation chip Loihi provides us with the tools to implement and test out the various neuromorphic features. The output provided by Loihi simulations is then compared to the output of classical simulations implemented in BMTK.

### 2.1. The Brain Modeling Toolkit (BMTK)

The BMTK is a python-based software package for creating and simulating large-scale neural networks. It supports models of different resolutions, namely, Biophysical Models, Point Models, Filter Models, and Population Models along with the use of the rich data sets of the Allen Cells Database (Lein et al., 2007; AIBS, 2020). It leverages the modeling file format SONATA which includes details on cell, connectivity and activity properties of a network along with being compatible with the neurophysiology data format Neurodata Without Borders (NWB), thus allowing easy access to a vast repertoire of experimental data.

In this study, we work with the Point Neuron Models with simulations supported by the BMTK module PointNet *via* NEST 2.16 (Kunkel et al., 2017; Linssen et al., 2018). For analysis and visualization, we use the HDF5 output format, underlying both SONATA and NWB's spike and time series storage.

The classical BMTK simulation are instantiated and run on a single node of Kamiak, the institutional high performance computing cluster. A typical Kamiak node contains 2 Intel Xeon E5-2660 v3 CPUs at 2.60 GHz, with 20 cores and 128–256 GB RAM (CIRC and WSU, 2021).

### 2.2. Loihi

Neuromorphic hardware inspired by the structure and functionality of the brain, envisioned to provide advantages such as low power consumption, high fault tolerance and massive parallelism for the next generation of computers, is called neuromorphic hardware. Toward the end of 2017, Intel Corporation unveiled its experimental neuromorphic chip called Loihi. We provide a summary of the platform here.

**Figure 2:**
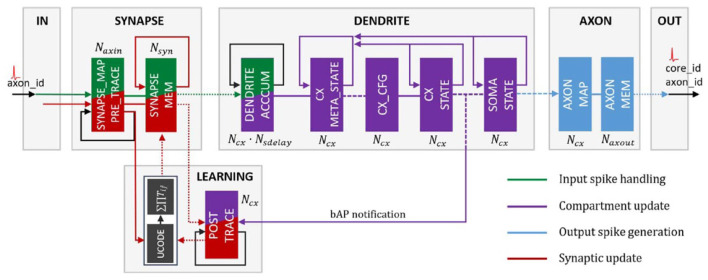
Loihi internal neuron model—Time multiplexed pipeline architecture of a neural unit (Figure 4 in Davies et al., 2018). Reproduced from WikiCommons (2018).

As of its 2020 rendition, the version on which these results were evaluated, Loihi is a 60-mm^2^ chip that implements 131,072 leaky-integrate-and-fire neurons. According to Davies et al. (2018), it uses an asynchronous spiking neural network (SNN), comprising of 128 neuromorphic cores, each with 1,024 neural computational units; 3 × 86 cores; along with several off-chip communication interfaces that provide connectivity to other chips. As Loihi advances the modeling of SNNs in silicon, it comprises of a large number of features necessary for their implementation viz., hierarchical connectivity, dendritic compartments, synaptic delays and synaptic learning rules. Each neuron is represented as a compartment in the Loihi architecture, i.e., it is designed to resemble an actual biological neuron model comprising of all the functional units (Figure 2). The SYNAPSE unit processes all the incoming spikes from the previous compartment/neuron and captures the synaptic weight from the memory. The DENDRITE unit updates the different state variables. The AXON unit generates the spike message to be carried ahead by the fan out cores. The LEARNING unit updates the synaptic weights based on a learning rule and is not used in this project.

The aim of this study is to establish the groundwork required to execute an ambitious plan of simulating about ~250,000 neurons with ~500M synapses in the future, which encapsulates much of the experimentally observed dynamics in the mouse visual cortex available to the AIBS, thus providing a close functional replica of the mouse visual cortex. Loihi's specialized hardware features hold promise for a real-time, low-powered version of such an implementation.

### 2.3. Leaky Integrate and Fire Model (LIF)

A typical neuron consists of a soma, dendrites, and a single axon. Neurons send signals along an axon to a dendrite through junctions called synapses. The classical Leaky Integrate and Fire (LIF) equation (Gerstner and Kistler, 2002) is a point neuron model which reduces much of the neural geometry and dynamics in order to achieve computational efficiency. It is one of the simplest and rather efficient representations of the dynamics of the neuron, while still providing reasonable approximation of biological neural dynamics for *some* classes of neurons (Teeter et al., 2018). It is stated mathematically as:

(1) $$\begin{array}{l} V^{\prime }\left(t\right)=\frac{1}{C}\left[I_{e}\left(t\right)-\frac{1}{R}\left(V\left(t\right)-E_{L}\right)\right] \end{array}$$

(2) $$\begin{array}{l} V\left(t\right)\leftarrow V_{r},\text{ if }V\left(t\right)>\Theta \end{array}$$

where,

$$\begin{array}{l} V\left(t\right)=\text{membrane potential (state)}\\ \;C=\text{membrane capacitance (parameter)}\\ \;R=\text{membrane resistance (parameter)}\\ \;E_{L}=\text{resting potential (parameter)}\\ \;I_{e}=\text{trans-membrane current (control and state)}\\ \;V_{r}=\text{reset membrane potential}\\ \;\Theta =\text{firing threshold} \end{array}$$

Here, ′ = *d*/*dt*, *t* is time in *ms*, the membrane potential *V*(*t*) of the neuron is in mV. These specific physical units are followed based on what the AIBS datasets use to define the respective physiology measurements. A LIF neuron fires when *V*(*t*) > Θ, i.e., the membrane potential exceeds the firing threshold Θ and subsequently the membrane potential is set to a reset value *V*_*r*_.

The classical LIF model (point generalized LIF) has been shown to match well the dynamics of some mouse neurons under a variety of conditions (Teeter et al., 2018), as listed in the Allen Cell Types Database (Lein et al., 2007). In addition, this model matches the LIF abstraction in Loihi to some extent (as Loihi uses discrete time discrete state dynamics to emulate the continuous time continuous state dynamics of the model). Thus, we work with this model throughout this study to establish the basis for comparison for the two platforms, determine how closely such a discrete dynamical system can get to simulations of a continuous dynamical system, validate the neuromorphic implementation against the ground truth of a standard implementation, and provide evidence that our neuromorphic platform performs more efficiently.

### 2.4. Loihi LIF Model

In an SNN, spiking neurons form the primary processing elements. The individual neurons are connected through junctions called synapses and interact with each other through single-bit events called spikes. Each spike train can be represented as a list of event times, e.g., as a sum of Dirac delta functions $\sigma \left(t\right)=\sum_{i}\delta \left(t-t_{i}\right)$ where *t*_*i*_ is the time of the *i*-th spike.

Since Loihi encapsulates the working of an SNN, one of the computational models it implements is a variation of the LIF model based on two internal state variables : the synaptic current and the membrane potential (Davies et al., 2018).

(3) $$\begin{array}{l} u\left(t\right)=\underset{j}{\sum }w_{j}\left(\alpha_{j}*\sigma_{j}\right)\left(t\right)+b \end{array}$$

(4) $$\begin{array}{l} v^{\prime }\left(t\right)=-\frac{1}{\tau_{v}}v\left(t\right)+u\left(t\right) \end{array}$$

(5) $$\begin{array}{l} v\left(t\right)\leftarrow 0,\text{ if }v\left(t\right)>\theta \end{array}$$

where,

$$\begin{array}{l} v\left(t\right)=\text{membrane potential}\\ u\left(t\right)=\text{synaptic current}\\ \;w=\text{synaptic weight}\\ \;\alpha =\text{synaptic response function}\\ \;b=\text{constant bias current}\\ \;\tau_{v}=\text{time constant}\\ \;\theta =\text{firing threshold} \end{array}$$

A neuron sends out a spike when its membrane potential exceeds its firing threshold θ, i.e., *v*(*t*) > θ. After a spike occurs, *v*(*t*) is reset to 0. As in the classical LIF model, here ′ = *d*/*dt*. However, time and membrane potential values here are in arbitrary units.

Loihi follows a fixed-size discrete time-step model, similar to an explicit Euler integration scheme, where the time steps relate to the algorithmic time of the computation. This algorithmic time may differ from the hardware execution time. Moreover, to increase the efficiency of the chip, specific bit-size constraints are imposed on the state variables. We discuss the ones relevant for the LIF model implementation in the following section.

## 3. Methods

### 3.1. Model Setup and Integration

The classical LIF model as represented in Equations (1) and (2) can be rewritten as :

(6) $$\begin{array}{l} V^{\prime }\left(t\right)=-\frac{1}{\tau_{v}}V\left(t\right)+\frac{1}{C}\left[I_{e}\left(t\right)+\frac{1}{R}E_{L}\right] \end{array}$$

where τ_*v*_ = *RC* is membrane time constant of the neuron.

For a non-homogeneous linear differential equation,

(7) $$\begin{array}{l} \frac{df}{dt}=af+g \end{array}$$

the solution is given by the “variation of constants” method as :

$$\begin{array}{l} f\left(t\right)=e^{at}\int_{0}^{t}g\left(s\right)e^{-as}ds \end{array}$$

Comparing Equation (6) to Equation (7), we have,

$$\begin{array}{l} a=\frac{1}{\tau_{v}}\\ f=V\left(t\right)\\ g=\frac{1}{C}\left(I_{e}\right)+\frac{1}{\tau_{v}}\left(E_{L}\right) \end{array}$$

Here, the postsynaptic current *I*_*e*_ is in the form of an exponent function. However, calculating the above integral at every step i.e., at all grid points *t*_*i*_ ≤ *t* proves to be quite expensive.

BMTK uses NEST as backend to implement the above membrane potential dynamics. To avoid the expensive computations, NEST chooses to use the linear exact integration method (Rotter and Diesmann, 1999), given below as follows :

Equation (6) is rewritten as a multidimensional homogeneous differential equation:

(8) $$\begin{array}{l} \frac{d}{dt}y=Ay \end{array}$$

where,

$$\begin{array}{l} A=\left(\begin{array}{cccccc} a_{n} & a_{n-1} & \cdots & \cdots & a_{1} & 0\\ 1 & 0 & \cdots & 0 & 0 & 0\\ 0 & \ddots & \ddots & \vdots & \vdots & \vdots \\ \vdots & \ddots & \ddots & 0 & 0 & 0\\ 0 & 0 & \ddots & 1 & 0 & 0\\ 0 & 0 & \cdots & 0 & \frac{1}{C} & \frac{1}{-\tau } \end{array}\right) \end{array}$$

The solution is given by :

(9) $$\begin{array}{l} y\left(t\right)=e^{At}y_{0} \end{array}$$

(10) $$\begin{array}{l} y_{t+h}=y\left(t+h\right)=e^{A\left(t+h\right)}y\left(0\right)=e^{Ah}\cdot y_{t} \end{array}$$

for a fixed time-step *h*. It saves exorbitant computations since each evaluated step involves multiplication only, and intermediate steps between events do not have to be computed.

#### 3.1.1. Mapping Between BMTK and Loihi Models

In this section, we illustrate the primary step of implementing the BMTK-NEST LIF integration with the Loihi dynamic computational model. Loihi follows a discrete-state, discrete-time computational model, similar to an explicit Euler integration scheme. This allows it more flexibility for integrating non-linear neural model, but, and unlike NEST's exact integration method, Loihi's engine accumulates errors at each time step. The time steps in the Loihi model relate to the algorithmic time of the computation which may differ from hardware execution time. Following the linear exact numerics in the NEST implementation, we implement our model in the Loihi discrete setting using the forward Euler method for guidance, as discussed below.

**Step 1**

First, we rewrite the standard LIF model in Equation (1) to resemble the Loihi form as given in Equation (4). Since Loihi parameters are unit-less, we introduce a re-scaling parameter *V*_*s*_, which converts standard physical units used in BMTK to Loihi units.

As we compare Equations (2) and (5), it can be seen that for BMTK the membrane potential reset value is set to *V*_*r*_ whereas it is set to zero for Loihi. To account for that, we shift the BMTK representation by *V*_*r*_. Thus, the forward transformation from BMTK to Loihi looks as follows :

(11) $$\begin{array}{l} v=\left(V-V_{r}\right)/V_{s} \end{array}$$

which produces an inverse transformation, to arrive back at the BMTK values, given by :

(12) $$\begin{array}{l} V=v\cdot V_{s}+V_{r} \end{array}$$

**Step 2**

Substituting the expression in (12) in (1) and isolating *v*, we get :

(13) $$\begin{array}{l} V^{\prime }\left(t\right)=\frac{1}{C}\left[I_{e}\left(t\right)-\frac{1}{R}\left(V\left(t\right)-E_{L}\right)\right]\;|V=vV_{s}+V_{r}\;\Rightarrow \; \end{array}$$

(14) $$\begin{array}{l} v^{\prime }\left(t\right)V_{s}=-\frac{1}{RC}\left(vV_{s}+V_{r}-E_{L}\right)+\frac{1}{C}I_{e}\left(t\right)\;|/V_{s}\;\Rightarrow \; \end{array}$$

(15) $$\begin{array}{l} v^{\prime }\left(t\right)=-\frac{1}{\tau_{v}}v\left(t\right)+\frac{1}{\tau_{v}}\frac{E_{L}-V_{r}}{V_{s}}+\frac{1}{C}\frac{I_{e}}{V_{s}} \end{array}$$

(16) $$\begin{array}{l} =-\frac{1}{\tau_{v}}v\left(t\right)+u\left(t\right) \end{array}$$

with

(17) $$\begin{array}{l} v\left(t\right)=\frac{V\left(t\right)-V_{r}}{V_{s}}, \end{array}$$

(18) $$\begin{array}{l} u\left(t\right)=\frac{1}{CV_{s}}I_{e}\left(t\right)+\frac{1}{\tau_{v}}\frac{E_{L}-V_{r}}{V_{s}}, \end{array}$$

(19) $$\begin{array}{l} \tau_{v}=RC, \end{array}$$

(20) $$\begin{array}{l} \theta =\frac{\Theta -V_{r}}{V_{s}} \end{array}$$

Here, we reintroduce the LIF threshold Θ and the corresponding Loihi threshold θ in Equation (20), which is derived from Θ by the same shift and re-scaling that converted *V* to *v*.

To reiterate, Loihi implements the continuous LIF as a discrete finite state machine model (Jin et al., 2008; Mikaitis et al., 2018) implemented in silicon. The actual computation is similar to a forward Euler scheme with some peculiarities reflecting engineering design trade-offs. Specifically, the *v*(*t*) state evolves on-chip according to the update rule,

(21) $$\begin{array}{l} v\left(t+1\right)=v\left(t\right)\left[1-\frac{\delta_{v}}{2^{12}}\right]+b+u\left(t\right) \end{array}$$

where δ_*v*_ is the membrane potential decay constant and *b* is the constant bias current listed in Equation (3).

**Step 3**

Using the forward Euler method :

$$\begin{array}{l} y_{n+1}=y_{n}+f\left(t_{n},y_{n}\right).dt \end{array}$$

where *y*_*n*+1_ = *y*(*t*_*n*+1_) and *t*_*n*+1_ = *t*_*n*_ + *dt* for a fixed time-step *dt*, we transform the classical LIF model into a form followed in Equation (21). Thus, transforming the LIF model into the discrete form and grouping terms to match the Loihi integration (9) yields the following :

(22) $$\begin{array}{l} \frac{v\left(t+dt\right)-v\left(t\right)}{dt}=-\frac{1}{\tau_{v}}v\left(t\right)+u\left(t\right) \end{array}$$

(23) $$\begin{array}{l} \;\Rightarrow \;v\left(t+dt\right)=v\left(t\right)\left(1-\frac{dt}{\tau_{v}}\right)+u\left(t\right)dt \end{array}$$

where *dt* is the fixed time-step with which we can adjust the temporal precision of the Euler integration scheme.

In order to equate the Loihi computation (21) with the Euler scheme (23), we use *dt* with units *ms/Loihi timestep* i.e., 1 BMTK millisecond per Loihi timestep. Thus, comparing Equations (21) and (23) defines the Loihi voltage decay parameter δ_*v*_ in terms of the timestep *dt*, i.e.,

(24) $$\begin{array}{l} \left(2^{12}-\delta_{v}\right)\;2^{-12}=\left(1-\frac{dt}{\tau_{v}}\right) \end{array}$$

(25) $$\begin{array}{l} \;\Rightarrow \;\delta_{v}=\frac{dt}{\tau_{v}}\;2^{12}=\frac{dt}{RC}\;2^{12} \end{array}$$

#### 3.1.2. Bit Constraints

Given its discrete setting, there are specific bit-size constraints that Loihi imposes on the state variables and parameters. State variables—membrane potential and current—are allotted ±23 bits each. The membrane potential decay constant δ_*v*_ is allotted 12 bits and the membrane potential threshold is assigned 17 bits interpreted as the 17 high bits of a 23 bit word to match the state variables size. Details on other parameters can be found under Table 1 in Davies et al. (2018) and Table 6 in Michaelis et al. (2021).

### 3.2. Validation Methods

#### 3.2.1. Data

The data used here is provided by the Allen Mouse Brain Atlas (Lein et al., 2007; AIBS, 2020), which is a survey of single cells from the mouse brain, obtained *via* intracellular electrophysiological recordings done through a highly standardized process. We focus on neurons of different types with available GLIF parameters. The data used can be accessed in the Allen Cell Types Database. Our LIF model is implemented and simulated on BMTK based on this data, and these simulations form the ground truth for validating the Loihi implementations.

The datasets used for the simulations in this work can be found in our Github repository (Dey, 2022).

#### 3.2.2. Cost Functions

To quantify the error between the BMTK and Loihi membrane potential values, we use two related cost functions: the Root Mean Square Error (RMSE) and the Pearson correlation coefficient (*r*) with values as follows :

(26) $$\begin{array}{l} RMSE=\sqrt{\frac{1}{n}\overset{n}{\underset{i=1}{\sum }}{\left(y_{L}^{i}-y_{B}^{i}\right)}^{2}} \end{array}$$

where,

$$\begin{array}{l} \;i=\text{index of data point}\\ y_{L}=\text{transformed Loihi values}\\ y_{B}=\text{original BMTK values}\\ \;n=\text{number of data points} \end{array}$$

and

(27) $$\begin{array}{l} r=\frac{\sum_{i=1}^{n}\left(y_{L}^{i}-\bar{y_{L}}\right)\left(y_{B}^{i}-\bar{y_{B}}\right)}{\sqrt{\sum_{i=1}^{n}{\left(y_{L}^{i}-\bar{y_{L}}\right)}^{2}\sum_{i=1}^{n}{\left(y_{B}^{i}-\bar{y_{B}}\right)}^{2}}} \end{array}$$

where,

$$\begin{array}{l} \bar{y_{L}}=\text{mean of the transformed Loihi values}\\ \bar{y_{B}}=\text{mean of the original BMTK values} \end{array}$$

#### 3.2.3. Other Methods

Since BMTK and Loihi run on two different computing environments, visual comparisons in graphs are helpful for diagnostics of discrepancies that may be obscured in the single numbers reported by the cost function. They also contribute to assess the level of similarity between the two implementations.

We compare the simulation dynamics for both implementations based on the following:

**- Distribution Function:** We compare the distributions of attained state values in the two cases. We use density plot as a representation of those distributions, thus allowing us to compare the two implementations in terms of concentration and spread of the values and provide a basis for comparing the collective dynamics of the implementations.
**- Raster Plot:** We evaluate the membrane potential response at each time-step. The X-axis represents the membrane potential and the Y-axis represents the time-step. Raster plot helps to visually communicate similarities between the BMTK and Loihi states, and highlight potential state-localized difference in the dynamics at each step which may otherwise be lost in the average error measures.
**- Scatter Plot:** For examining association between the two implementations, we use color-coded scatter plots identifying the correlation relationships. We add a trend line to illustrate the strength of the relationship and pin down the outliers to improve the simulation results. Since we anticipate an almost perfect linear relationship, we quantify the match with its Pearson correlation coefficient.

## 4. Results

In order to lay the groundwork for simulating a network of over 250,000 neurons with a connectivity of over 500M synapses in the neuromorphic hardware, we begin by ensuring a high quality replication of individual neural and smaller network models. The replication performance here is evaluated based on membrane potential and current responses, the two state variables. We conjecture that securing a good replica for smaller models will ensure that parameters can be calibrated correctly and thus can be carried forward for the bigger networks needed in biological context (Herz et al., 2006; Gutzen et al., 2018; Trensch et al., 2018).

We begin our work on a single-neuron network^1^ sub-threshold dynamics driven by both bias current and external spikes to ensure Loihi is able to handle both stimuli efficiently. Our test suite consists of LIF models based on 20 different parameter sets. We perform rigorous analysis of our results based on various statistical measures and visualizations to demonstrate that we have replication of high quality. It is important to restate here that we test our results based on neurons with different morphologies and biophysics, which attribute to the different parameter sets.

### 4.1. Simulations of a Single Neuron

We begin by simulating a single-neuron network in BMTK. The simulation is run for 500 *ms*. The classical parameters are translated to Loihi values and the corresponding LIF model is implemented as an one-neuron SNN executed for 500/*dt* time steps in Loihi. The simulations are driven either by bias current or external spikes.

In the Loihi network, neurons are denoted by compartments. The compartment dynamics are hardware-constrained and determined by the parameters *bias current mantissa, membrane potential threshold, membrane potential decay*, and *current decay*. It is worth iterating here that the membrane potential values in Loihi are unit-less as opposed to the BMTK values which are assigned units of millivolts (mV) and milliseconds (ms) based on the AIBS datasets.

**Figure 3:**
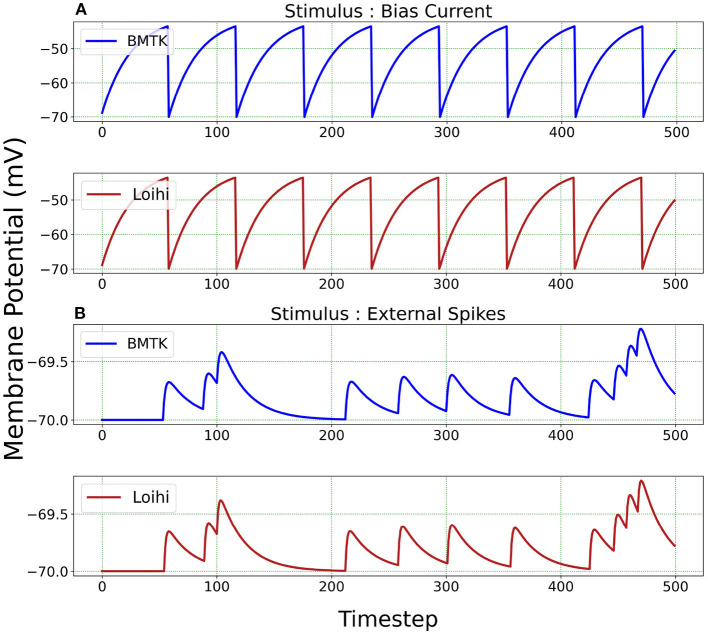
Membrane potential response for single-neuron network based on two different neuron parameters. **(A)** Simulation is driven by bias current. **(B)** Simulation is driven by external spikes.

We test the precision of our replication, both qualitatively and quantitatively, for all 20 parameters sets and find that the results are consistent with the ones described below. In Figure 3, we illustrate the implementations achieved through bias current and external spikes on two different parameter sets (Table 1). The remaining 18 parameter sets can be found in our Github page (Dey, 2022). The parameters in the BMTK platform are mapped to Loihi using the transformations described in Section 3.1.1 with respect to the bit constraints described in Section 3.1.2.

**Table 1:** Parameter set for LIF models.

**Parameters**	**Dataset (1)**	**Dataset (2)**	**Units**
Membrane time constant	25.0	22.0	*ms*
Membrane potential threshold	−43.0	−43.0	*mV*
Resting potential	−70.0	−70.0	*mV*
Voltage reset	−70.0	−70.0	*mV*
Current	200.0	0.0	*pA*
Membrane capacitance	170.21	170.0	*pF*

It is to be noted here that stimulus bias current acts as one of the parameters of the LIF model and hence is mapped into Loihi according to Equation (18). When stimulating with external spikes as stimulus, we make use of the fixed-time step *dt* that we introduce in Equation (23). Here, the external spike-times are in “*ms*” and we assign unit “ms/Loihi time-step” to *dt*. Thus, the external spike-times are scaled as spike-time/*dt* and then injected into a Loihi neural unit for each time-point, with *dt* guiding the temporal precision scale. Table 2 shows the external spike-times used in the simulations, which are generated by five spike sources using a random Poisson spike generator with a max firing rate of 5Hz and then frozen to stimulate the different models in both BMTK and Loihi.

**Table 2:** External spike-time values.

**Source**	**Spike-times (ms)**
0	446
1	355
2	53, 258, 300, 424, 457
3	88, 466
4	100, 212

For a qualitative comparison, it can be seen from Figure 3 that Loihi implementations simulate BMTK results very closely. We have close correspondence in terms of spike frequency, spike amplitude, and response values. Since Loihi membrane potential values are unit-less, we map them back to BMTK values (mV, ms) before performing the comparison. The inverse mapping from Loihi to BMTK is performed based on Equation (12), i.e.,

$$\begin{array}{l} V=v\cdot V_{s}+V_{r} \end{array}$$

We perform a quantitative assessment of the replication using RMSE and correlation coefficient between the values obtained from the two platforms. As seen from Table 3, the values are highly correlated with a relatively small RMSE.

**Table 3:** Correlation and RMSE between BMTK and Loihi membrane potential values.

**Stimulus**	**Correlation**	**RMSE**
Bias current	0.999992	1.1374 × 10^−4^ mV/*ms*
External spikes	0.999942	4.208 × 10^−5^ mV/*ms*

**Figure 4:**
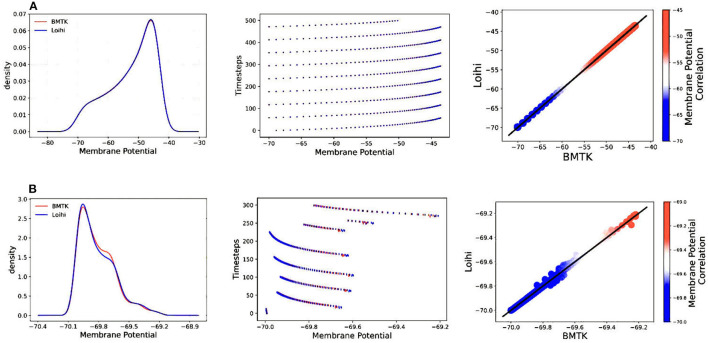
Validation plots for simulations based on two different stimuli—**(A)** Validation plots for bias current stimulus **(B)** Validation plots for external spike stimulus, based on the Distribution Function, Raster Plot, and Scatter Plot, respectively.

Figure 4 illustrates the comparison of Loihi implementations against the BMTK implementations for the two different stimuli using various graphing data—(a) Distribution function approximating the membrane potential dynamics, (b) Raster plot of the spiking network activity, (c) Scatter Plot highlighting the positive coefficient between the two implementations. These visualizations help us track discrepancies which might remain unobserved based on single quantitative averages given by the cost function or the correlation coefficient.

### 4.2. Simulation Using Varied Precision

As already stated, Loihi follows a fixed-size discrete time-step model along with bit-size constraints for the different parameters. Thus, we examine how the numerics of Loihi affect its ability to faithfully implement neuron models. More precisely, we investigate how changing the precision of the time scale and the neuron state values affects the accuracy of the simulations. We explore this property for the two state variables—membrane potential and current.

**Figure 5:**
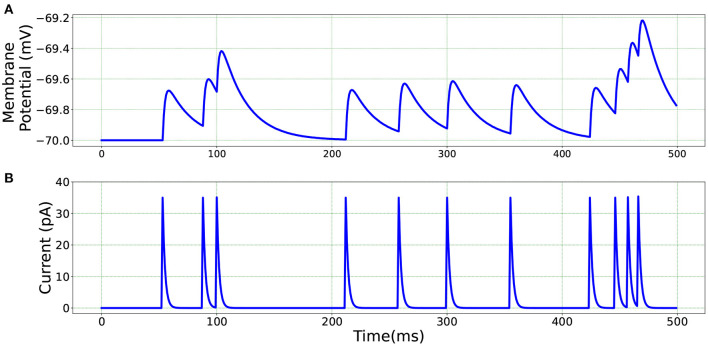
Single neuron model in BMTK—**(A)** Membrane potential response. **(B)** Current response.

Figure 5 illustrates the membrane potential and current responses of a single neuron model in BMTK which form the basis of our comparison for the results below.

#### 4.2.1. Simulation Using Varied Temporal Precision

For Loihi's fixed simulation time-step, we assign different time units to each step and test the corresponding simulation precision. This is achieved through the “*dt*” parameters available in our equations while transforming the classical LIF model to Loihi neural model. It enables us to experiment with several time units (Hopkins and Furber, 2015). Following Equation (24), the change of a time-step while working with the Loihi neural model necessitates a corresponding variation of the time constant “τ_*v*_” to yield the desired results.

**Figure 6:**
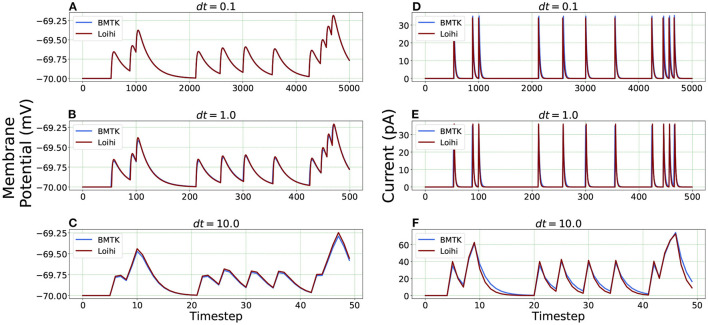
Comparison of membrane potential and current plots with different temporal precisions in Loihi. Membrane potential plots are on the left with **(A)** *dt* = 0.1 **(B)** *dt* = 1.0 **(C)** *dt* = 10.0. Current plots are on the right with **(D)** *dt* = 0.1 **(E)** *dt* = 1.0 **(F)** *dt* = 10.0. For *dt* = 10.0, number of time-steps are 50 and for *dt* = 0.1, number of time-steps are 5,000.

We check the results for *dt* = 0.1, 1.0 and 10.0(*ms*/*timestep*). As mentioned earlier, we run the simulation for 500 *ms*, thus the corresponding number of time steps in Loihi for *dt* = 0.1 and *dt* = 10.0 becomes 5000 and 50 respectively, and for *dt* = 1.0 it remains at 500, (i.e., 500/*dt* for each *dt*). Figure 6 illustrates the related Loihi simulation for membrane potential and current.

***Error comparison for temporal precision*** Unlike continuous analysis in which the error decreases monotonically with *dt*, Loihi's discrete-time, discrete-state simulation dynamics suggests that there may be an optimal *dt* which minimizes the LIF dynamics error.

**Figure 7:**
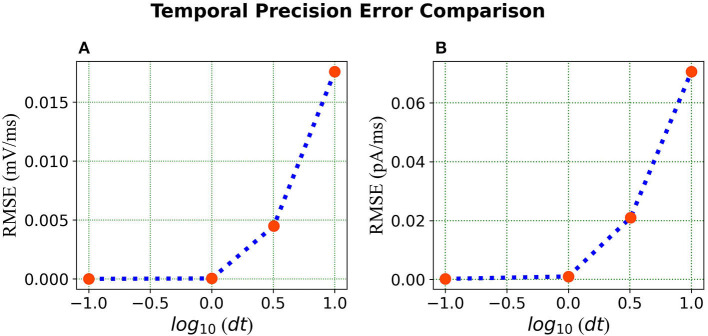
Error comparison for different temporal precisions—**(A)** Membrane potential error. **(B)** Current error. In both panels, the RMSE for the corresponding state is plotted against the *log* of the temporal precision *dt*.

We compare the simulations in Loihi with different temporal precisions against the simulations in BMTK. We calculate the RMSE to be able to deduce the result. As can be seen from Figure 7, the error is lowest when 1 *ms* of simulation time in BMTK equates to 1 time-step in Loihi for membrane potential and current. Thus, for the LIF model simulations, representing 1*ms* with a Loihi hardware time step provides the best match between the two simulations. As to using larger *dt* for efficiency, panels (C) and (F) clearly show that large time steps (larger than the synaptic time constant in this case) significantly degrade the quality of simulations.

It should be noted that the selected simulation timestep *dt* affects the range of physical time constants τ_*v*_ that can be represented in Loihi. Since $\delta_{v}=\frac{dt}{\tau_{v}}\;2^{12}$ (Equation 24), then $\tau_{v}=\frac{dt}{\delta_{v}}\;2^{12}$. In Loihi, $\delta_{v}\in \left[1,2^{12}\right]$ (stored as a 12-bit word, with 0 representing $\delta_{v}=2^{12}$). Hence, $\tau_{v}\in \left[1,2^{12}\right]dt$, that is, the highest physical time constant that can be represented is $\tau_{v}=2^{12}dt\approx 4,100dt$. This is not a big constraint for *dt* = 1*ms*, but e.g., a higher simulation precision of *dt* = 0.01*ms* can be performed only for neurons with time constants τ_*v*_ <41*ms*, which already excludes some models found in the Allen Institute's Cell Types Database. This shortcoming of this Loihi 1 platform is being addressed by Intel in subsequent hardware like Loihi 2 (Intel, 2022), and the new Lava SDK. Similarly affected are potential spike propagation delays (not used here). Loihi supports ranges from 1 to 62 time steps, which translate to *dt* to 62*dt ms* of physical time. This is a minor constraint for *dt* = 1*ms*, but quickly becomes a significant constraint for short *dt*.

#### 4.2.2. Simulation Using Varied Voltage Precision

**Figure 8:**
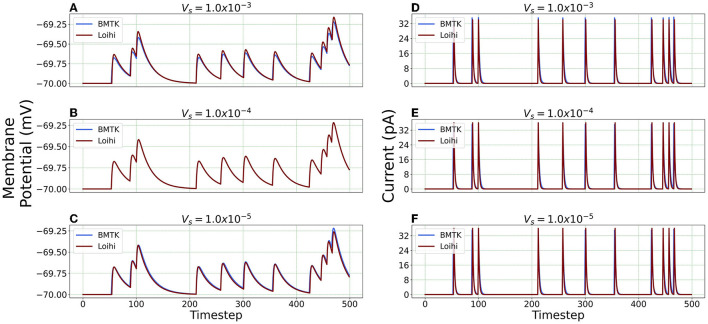
Comparison of membrane potential and current plots with different voltage precisions. Membrane potential plots are on the left with **(A)** $V_{s}=1.0\times 10^{-3}$ **(B)** $V_{s}=1.0\times 10^{-4}$ **(C)** $V_{s}=1.0\times 10^{-5}.$ Current plots are on the right with **(D)** $V_{s}=1.0\times 10^{-3}$ **(E)** $V_{s}=1.0\times 10^{-4}$ **(F)** $V_{s}=1.0\times 10^{-5}$.

We repeat the precision study by changing the voltage precision values using the re-scaling parameter *V*_*s*_. To check different precision results, we try 1K/mV, 10K/mV and 100K/mV (state level/mV) by using $V_{s}=1.0\times 10^{-3},1.0\times 10^{-4}$ and 1.0 × 10^−5^ respectively. Figure 8 illustrates the neuron state simulations based on different voltage precisions.

**Figure 9:**
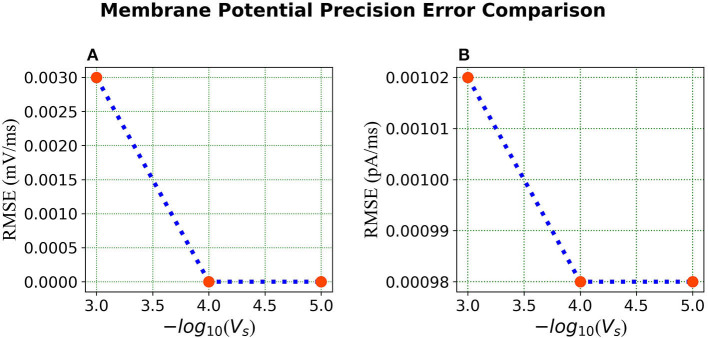
Error comparison for different membrane potential precisions—**(A)** Membrane potential error. **(B)** Current error. In both panels, the RMSE for the corresponding state is plotted against the –*log* of the voltage scale *V*_*s*_.

***Error Comparison for Voltage Precision*** As can be seen from Figure 9, for membrane potential—error decreases significantly as the precision increases from $V_{s}=1.0\times 10^{-3}$ to $V_{s}=1.0\times 10^{-4}$. However, the error is extremely small for the current simulation and remains the same for $V_{s}=1.0\times 10^{-4}$ and $V_{s}=1.0\times 10^{-5}$.

#### 4.2.3. Effects of State Precision on Simulations

In conclusion, the effect of precision on both scales depends on the model parameters and the information needing to be preserved. However, there are important performance differences. Increased voltage precision is essentially free, as it does not tax the hardware resources any further, and the sole risk is from computation overflow in cases of the Loihi voltage state nearing the capacity of the voltage register. Increased time precision on the other hand has two important drawbacks: it increases simulation time (proportionately to increased precision), and it decreases the range of voltage decay timescales that can be represented (again, proportionately to increased precision). Thus, the choice of simulation time step and corresponding precision should be weighed against these tradeoffs.

### 4.3. Simulation of Different Neuron Classes

After establishing and verifying the calibrated Loihi parameters for a single neuron, we extend our simulation to an ensemble of neurons comprising of different neuron classes with varying parameters.

**Figure 10:**
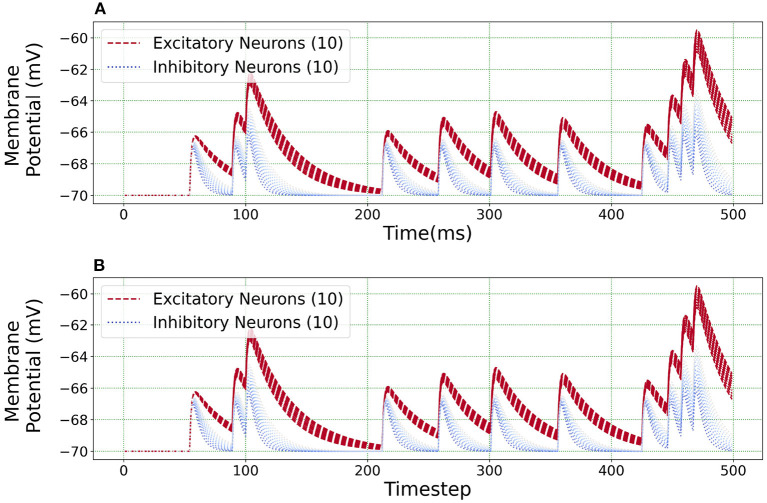
Loihi replicates various neuron class responses of BMTK. **(A)** BMTK simulation of 20 neuron classes. **(B)** Loihi simulation of 20 neuron classes.

Figure 10 illustrates an equivalent simulation for 20 different neuron classes between BMTK and Loihi indicating that Loihi is capable of emulating BMTK results in spite of varying parameters. Here, we found an average correlation of 0.99985 with an RMSE of 0.57 × 10^−4^ mV/*ms* (Table 4). This also validates the fact that the calibration of parameters for a single neuron done earlier is valid.

**Table 4:** Correlation and RMSE for different neuron classes.

**Neuron class**	**Correlation**	**RMSE**
Excitatory	0.999989	0.532 × 10^−4^ mV/*ms*
Inhibitory	0.999982	0.612 × 10^−4^ mV/*ms*

**Figure 11:**
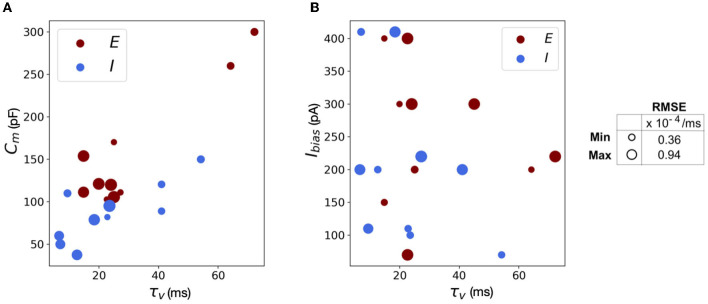
Scatter plots showing the range of parameters for the 20 neurons classes comprising of both excitatory and inhibitory neurons grouped by RMSE of the simulations. **(A)** Scatter plot for membrane capacitance (*C*_*m*_) vs. membrane time constant (τ_*v*_). **(B)** Scatter plot for bias current (*I*_*bias*_) vs. membrane time constant (τ_*v*_). The marker size is determined by the corresponding RMSE.

The scatter plots in Figure 11 capture the range of the parameters—Figure 11(A) *C*_*m*_ vs. τ_*v*_ and Figure 11(B) *I*_*bias*_ vs. τ_*v*_, in the (E)xcitatory and (I)nhibitory classes used for the simulations. The size of the markers represents RMSE errors for those models, with ranges as indicated on the legend. This lays the foundation for building more complicated networks encompassing different neuron classes.

We reiterate here that Loihi imposes certain bit constraints on the parameters. For instance, membrane potential threshold ranges from 0 to ± 2^23^, membrane time constant allows 0 to 2^12^ bits. The membrane capacitance is integrated with bias current (Equation 18) with bias mantissa allowed a range between [−2^12^, 2^12^] and bias exponent a range between [0, 7]. Thus, a good range of parameters can be mapped well into Loihi and a limit to the “exactness” can be attributed to the low-fixed-precision nature of Loihi as most state and configuration variables are in the range of 8–24 bits.

## 5. Conclusion and Future Work

Inspired by the brain, neuromorphic computing holds great potential in tackling tasks with extremely low power and high efficiency. Many large-scale efforts including the TrueNorth, SpiNNaker and BrainScaleS have been demonstrated as a tool for neural simulations, each replete with its own strengths and constraints. Fabricated with Intel's 14 nm technology, Loihi is a forward-looking and continuously evolving state-of-the-art architecture for modeling spiking neural networks in silicon. As opposed to its predecessors, Loihi encompasses a wide range of novel features such as hierarchical connectivity, dendritic compartments, synaptic delays and programming synaptic learning rule. These features, together with solid SDK support by Intel, and a growing research community, make Loihi an effective platform to explore a wealth of neuromorphic features in more detail than before.

In this work, we have demonstrated that Loihi is capable of replicating the continuous dynamics of point neuronal models with high degree of precision and does so with much greater efficiency in terms of time and energy. The work comes with its challenges as simulations built on the conventional chips cannot be trivially mapped to the neuromorphic platform as its architecture differs remarkably from the conventional hardware. Classical simulations from the Brain Modeling Toolkit (BMTK) developed by the Allen Institute of Brain Science (AIBS) serves as the foundation of our neuromorphic validation.

For comparison between the conventional and the neuromorphic platforms, we use both qualitative and quantitative measures. It can be seen that Loihi replicates BMTK very closely in terms of both membrane potential and current, the two state variables on which the Loihi LIF model evolves. We use different validation methods and quantitative measures to assess the equivalence and identify sets of parameters which maximize precision while retaining high performance levels. Furthermore, simulation results indicate Loihi is highly efficient in terms of speed and scalability as compared to BMTK.

This work demonstrates that classical simulations based on Generalized Leaky Integrate-and-Fire (GLIF) point neuronal models can be successfully replicated on Loihi with a reasonable degree of precision.

**Figure 12:**
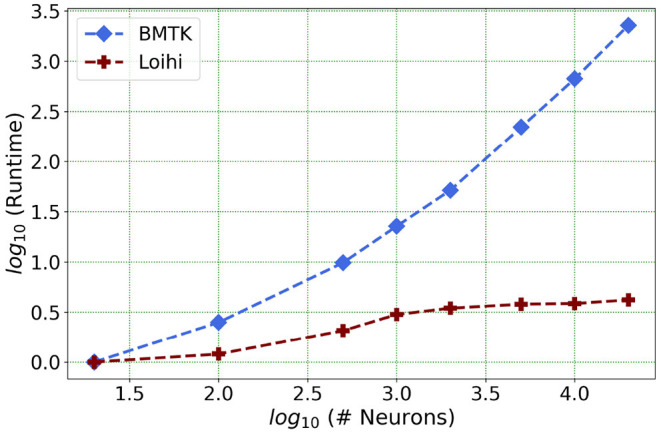
Performance comparison between BMTK and Loihi for network sizes ranging from 1 to 20,000 for the simulation of 500 *ms* of dynamics. The values for each curve are scaled by the respective smallest runtime. The Loihi runtime units are in “*milliseconds*” and BMTK runtime is in “*seconds*”.

Our future work is motivated by runtime performance comparisons for larger networks between the two platforms. As Loihi and BMTK are based on very different hardware systems that follow distinct dynamics and network-setup regimes, we use the runtime of the simulations to compare the performance of these implementations. As has been mentioned in the introduction, performance of Loihi far exceeds that of BMTK. Figure 12 compares the runtime of Loihi and BMTK, for running a network of randomly connected neurons with the same parameters. The network consists of excitatory and inhibitory neurons in a 1:1 ratio driven by bias current, with connection probability set at 0.1.

As can be seen from Figure 12 and Table 5, Loihi easily scales up to larger network sizes with a minuscule rise in runtime whereas for BMTK the increase is quite rapid. While both seem to exhibit a power-law scaling (string line on this graph), Loihi's scaling power is much smaller. It is also worth noting here that for Loihi the unit for the runtime are in “*milliseconds*” whereas for BMTK they are in “*seconds*”. Here we stop at 20,000 neurons as it can be inferred from the graph that increasing the network size would increase the time cost for BMTK substantially.

**Table 5:** Simulation runtime in Loihi and BMTK.

**Network size**	**Loihi time (ms)**	**BMTK time (s)**
20	2.52	0.12
100	3.03	0.3
500	5.21	1.13
1,000	7.56	2.72
5,000	9.57	26.47
10,000	9.73	80.45

**Figure 13:**
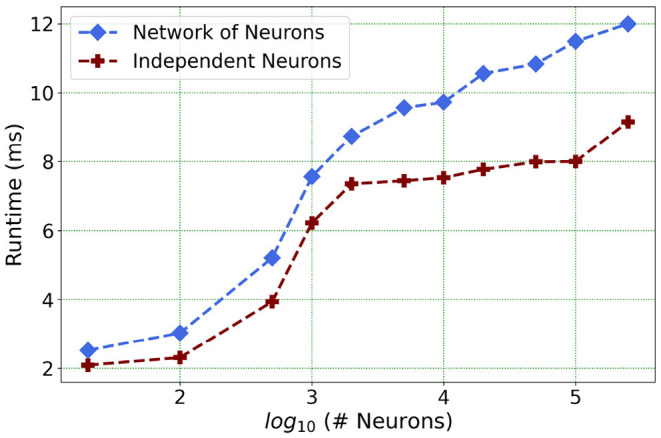
Loihi runtime for a network of upto 250K neurons for the simulation of 500 *ms* of dynamics.

Furthermore, following the above outcome, we extend our network size *in Loihi only* to 250K neurons in order to investigate what potential Loihi holds to execute the final goal of simulating about ~250,000 neurons with ~500M synapses in the future, a simulation scale comprising much of the experimentally observed dynamics in the mouse visual cortex available to the AIBS. We record our observations for a randomly connected network of neurons as well as an independent set of unconnected neurons. From Figure 13 and Table 6, we can infer that the runtime remains consistent with the above result, with the independent set of neurons completing the simulation marginally faster.

**Table 6:** Simulation runtime in Loihi for independent neurons vs. connected network.

**Network size**	**Connected network (ms)**	**Independent neurons (ms)**
20	2.52	2.09
100	3.03	2.31
500	5.21	3.94
1,000	7.56	6.22
5,000	9.57	7.35
10,000	9.73	7.53
50,000	10.84	7.98
100,000	11.49	8.00
250,000	11.98	9.16

This shows that Loihi performs well for connected networks, setting the stage for our main aim for neural simulations. Additionally, it also works well for independent set of neurons which contribute to solutions of problems that require on-chip parameter and meta-parameter searches, e.g., for Evolutionary Programming (Schuman et al., 2020).

We do not asses the state-based cost for these networks as their large sizes require multi-chip simulations which we expect to be better supported on Loihi 2 (Intel, 2022). Furthermore, other research groups have firmly established that we cannot expect exact replication of subthreshold network states between simulators except for few very simple small networks (van Albada et al., 2018; Crook et al., 2020). Thus, on the network level we need to develop cost functions that capture appropriate network activity details on different scales (e.g., average spike rates and correlations on the coarsest levels, as in van Albada et al., 2018).

In closing, we want to highlight that with the advent of Loihi 2 (Intel, 2022), we aim to address the limitations of the larger networks and carry out the next steps of our work in this new hardware. We are planning to investigate the full GLIF dynamics as we would have better support for more complex network topology and spiking dynamics. In addition, we hope to implement a connected network of 250K neurons with specific synaptic variables as available in the AIBS dataset. We also plan to investigate the control and performance of temporal precision choices. Till date, our limited conclusion for these cases is that ~ 1*ms* timestep is sufficient. This need not generalize to networks in which other precision may be needed, with corresponding tradeoffs to changes in the parameters. We intend to explore this question further. Lastly, we are working on performing a sensitivity analysis on the GLIF parameters to assess the robustness of the model.

## Data Availability Statement

The original contribution in the study are included in the article. The datasets used are available in https://github.com/srijanie03/bmtk_loihi_utils. Further inquiries can be directed to the corresponding author/s.

## Author Contributions

SD and AD contributed to conception and design of the study. SD performed the simulations and analysis and wrote the first draft of the manuscript. AD wrote parts of the manuscript, edited, and reviewed. All authors contributed to manuscript revision, read, and approved the submitted version.

## Funding

This work received support from WSU Vancouver Office of Research and Graduate Education to SD.

## Conflict of Interest

The authors declare that the research was conducted in the absence of any commercial or financial relationships that could be construed as a potential conflict of interest.

## Publisher's Note

All claims expressed in this article are solely those of the authors and do not necessarily represent those of their affiliated organizations, or those of the publisher, the editors and the reviewers. Any product that may be evaluated in this article, or claim that may be made by its manufacturer, is not guaranteed or endorsed by the publisher.

## Footnotes

^1^A network is the smallest executable structure in Loihi, hence the peculiar term *single-neuron network*.
