# Erratum: Mapping and validating a point neuron model on Intel's neuromorphic hardware Loihi

**DOI:** 10.3389/fninf.2022.1107838

**Published:** 2023-01-16

**Authors:** Frontiers Production Office

**Affiliations:** Frontiers Media SA, Lausanne, Switzerland

**Keywords:** neuromorphic computing, LIF models, neural simulations, validation, performance analysis

Due to a technical error, in the original publication, the journal-level bibliographic information presented for this Frontiers in Neuroinformatics article was incorrect and referred erroneously to the journal Frontiers in Neuroscience. The DOI was also incorrect and has been amended as per the journal requirements.

The publisher apologizes for this mistake. The original article has been updated.

